# Venous stroke–a stroke subtype that should not be ignored

**DOI:** 10.3389/fneur.2022.1019671

**Published:** 2022-10-06

**Authors:** Yifan Zhou, Huimin Jiang, Huimin Wei, Lu Liu, Chen Zhou, Xunming Ji

**Affiliations:** ^1^Laboratory of Brain Disorders, Ministry of Science and Technology, Collaborative Innovation Center for Brain Disorders, Beijing Advanced Innovation Center for Big Data-Based Precision Medicine, Beijing Institute of Brain Disorders, Capital Medical University, Beijing, China; ^2^School of Engineering Medicine, Beijing Advanced Innovation Center for Big Data-Based Precision Medicine, Beihang University, Beijing, China; ^3^Department of Neurology, Xuanwu Hospital, Capital Medical University, Beijing, China; ^4^Department of Neurosurgery, Xuanwu Hospital, Capital Medical University, Beijing, China

**Keywords:** cerebrovascular disease, arterial stroke, cerebral venous thrombosis, dural sinus thrombosis, venous stroke

## Abstract

Based on the etiology, stroke can be classified into ischemic or hemorrhagic subtypes, which ranks second among the leading causes of death. Stroke is caused not only by arterial thrombosis but also by cerebral venous thrombosis. Arterial stroke is currently the main subtype of stroke, and research on this type has gradually improved. Venous thrombosis, the particular type, accounts for 0.5–1% of all strokes. Due to the lack of a full understanding of venous thrombosis, as well as its diverse clinical manifestations and neuroimaging features, there are often delays in admission for it, and it is easy to misdiagnose. The purpose of this study was to review the pathophysiology mechanisms and clinical features of arterial and venous thrombosis and to provide guidance for further research on the pathophysiological mechanism, clinical diagnosis, and treatment of venous thrombosis. This review summarizes the pathophysiological mechanisms, etiology, epidemiology, symptomatology, diagnosis, and treatment heterogeneity of venous thrombosis and compares it with arterial stroke. The aim is to provide a reference for a comprehensive understanding of venous thrombosis and a scientific understanding of various pathophysiological mechanisms and clinical features related to venous thrombosis, which will contribute to understanding the pathogenesis of intravenous stroke and provide insight into diagnosis, treatment, and prevention.

## Introduction

Stroke is a major cause of disability and mortality worldwide and the second leading cause of death in the United States (1, 2). The ischemic stroke accounts for the 87% of all cases, which results from the cerebral arteries occlusion due to thrombosis, atherosclerosis and platelets plug (3). Thrombosis also form in cerebral venous, which is termed as cerebral venous thrombosis(CVT), a particular type of cerebrovascular disease, characterized by intracerebral hemorrhage and infarction, associated with increased intracranial pressure due to cerebrospinal fluid absorption and cerebral venous drainage, accounting for 0.5–1% of strokes (4). To date, there are more extensive and comprehensive studies on arterial thrombosis, with few clinical and basic studies on venous thrombosis, which greatly limits our understanding of venous thrombosis and the development of related drugs. In this review, we summarize the etiology, pathogenesis, symptomatology, diagnosis, and treatment heterogeneity of venous thrombosis based on current studies.

## Molecular pathological hallmarks of ischemic stroke

### Hypoxia-an essential aspect of arterial stroke and cerebral venous thrombosis

**Figure 1:**
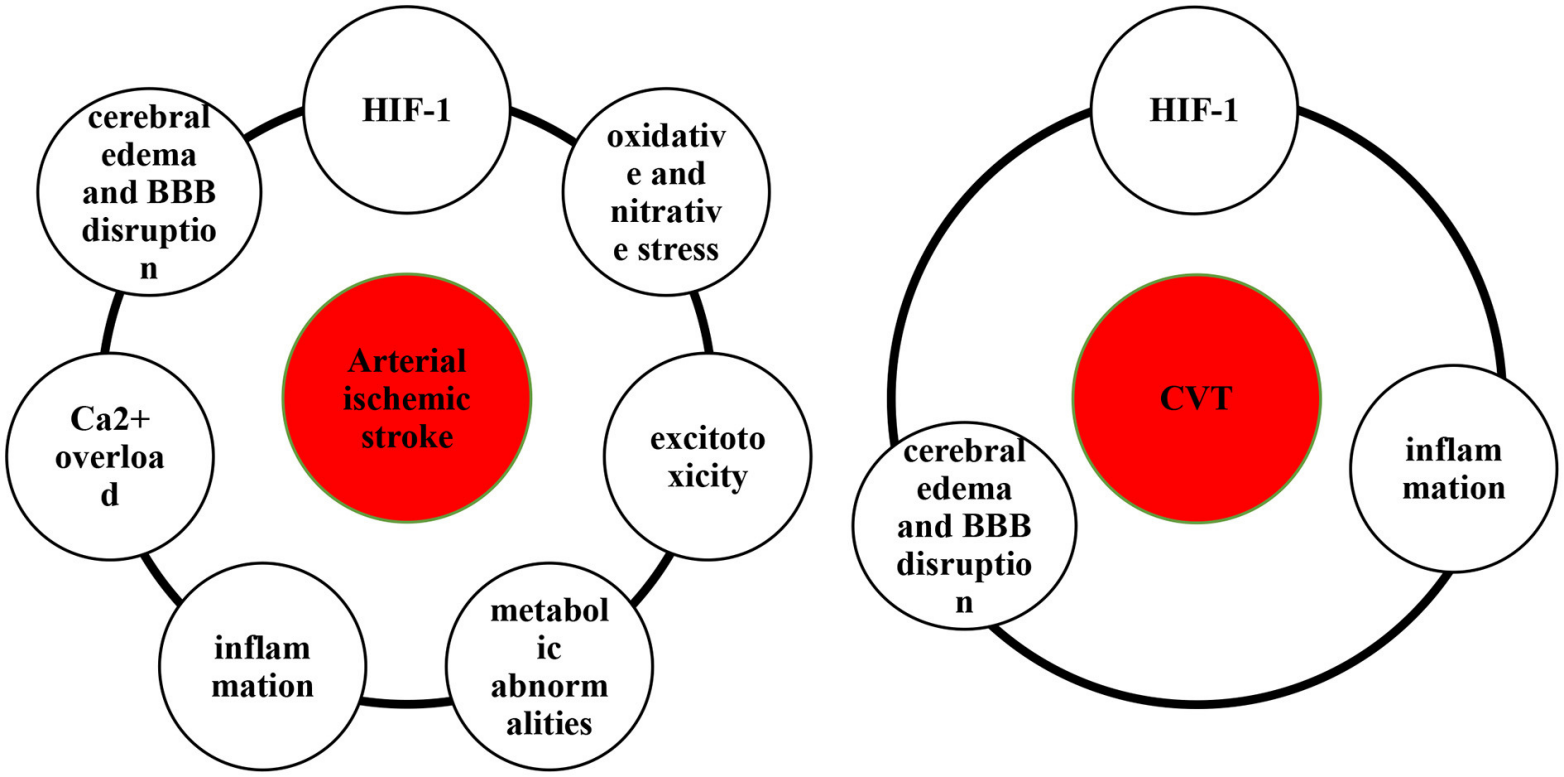
The molecular pathological hallmarks of arterial stroke and cerebral venous thrombosis.

Hypoxia and ischemia of the brain are key pathophysiological mechanisms of ischemic stroke (5, 6). Hypoxia caused by impaired blood circulation can be referred to as circulatory hypoxia, which are classified as ischemic hypoxia and congestive hypoxia. Ischemic hypoxia is caused by an impaired arterial blood supply, whereas congestive hypoxia results from an impaired venous return. Hypoxia is caused by the sudden decrease in cerebral blood flow due to ischemic stroke (5), resulting in hypoxia-inducible factor-1 (HIF-1) production (7), oxidative and nitrative stress (8, 9), excitotoxicity (10, 11), metabolic abnormalities (12, 13), inflammation (14, 15), Ca^2+^ overload (16), cerebral edema and blood–brain barrier (BBB) disruption (17) (Figure 1).

#### HIF-1

HIF-1, including HIF-1α and HIF-1β, is an important regulator of hypoxia in stroke and participates in the pathological process of stroke by regulating glucose metabolism, angiogenesis, erythropoiesis and cell survival (18–20). Li et al. found that HIF-1α attenuates neuronal apoptosis by upregulating erythropoietin in rats with cerebral ischemia (21). Moreover, under hypoxic conditions, HIF-1 dynamically regulates reactive oxygen species (ROS) production *via* the glycolytic pathway and tricarboxylic acid cycles (22). Using a model of permanent middle cerebral artery occlusion (MCAO), Marti et al. demonstrated that hypoxia-induced upregulation of HIF-1 and HIF-2 increases expression of vascular endothelial growth factor (VEGF), thereby promoting neoangiogenesis (23). However, bidirectional roles of HIF-1 in different cells. After stroke, HIF-1 induces production and secretion of cytokines and chemokines, which in turn exacerbate inflammatory injury (19, 24). Moreover, Koh et al. verified that hypoxia-triggered neutrophil migration is decreased in HIF-1α-deficient mice, which is an important factor in regulating brain injury (25). Wang et al. found that inhibition of HIF-1 expression reduces BBB damage (26). In general, the beneficial or detrimental effects of HIF-1 on stroke depend on the duration and severity of hypoxia in arterial stroke and CVT.

#### Oxidative and nitrative stress

Brain ischemia and hypoxia can produce oxygen free radicals (ORFs), lipid radicals, and reactive nitrogen species (RNS). When these free radicals exceed the endogenous scavenging capacity, cells undergo oxidative stress and nitrative stress, resulting in apoptosis, autophagy and necrosis (27). ORFs, such as ROS and nitric oxide synthase (NOS), are affected by nicotinamide adenine dinucleotide phosphate (NADPH) oxidase (28), mitochondrial depolarization (29), nitric oxide synthase (30) and xanthine oxidase (31), thus triggering a ROS surge. ROS not only mediate cellular structural damage but also alter vascular permeability, dilate diastolic blood vessels, disrupt the BBB and lead to focal brain damage (32). It was demonstrated that accumulation of lipid ROS leads to intracellular oxidative stress and iron death after stroke, which is a pathway of nonapoptotic cell death mediated by iron (33, 34). Nitric oxide (NO) is a type of RNS generated by NOS (35). Serrano-Ponz et al. recorded and collected the data and clinical history of patients (*n* = 76) with acute ischemic stroke and monitored certain parameters. They found that an increase in nitric oxide metabolite (NOx) levels from Day 1 to Day 2 was beneficial (odds ratio (OR) = 0.91) but that a sharp increase in NOx levels from Day 2 to Day 7 was detrimental, and levels of NOx were associated with an increase in infarct volume (36). However, studies on stress responses to intravenous stroke are lacking overall.

#### Excitotoxicity

Excitotoxicity occurs when oxygen is insufficient to support aerobic respiration of mitochondria after cerebral ischemia (5). Disorders of energy metabolism inhibit the activity of sodium-potassium adenosine triphosphate (ATP)ase, resulting in decreased ATP synthesis and an imbalance of ionic gradients inside and outside nerve cells (37). According to Pietrogrande et al., low oxygen post-conditioning limits excitotoxicity-induced neuronal death and promote neuronal survival after secondary injury (38). In addition, ischemic stroke is associated with release of glutamate in the brain (39). During ischemia, excessive release of glutamate results in cell death. Using an animal model of MCAO, Campos et al. showed that activation of glutamate oxaloacetate transaminase inhibits the increase in glutamate after cerebral ischemia (40). Infarct size, edema volume, and sensorimotor deficits are significantly reduced as a result of the activation of glutamate oxaloacetate transaminase (40, 41). Fang et al. examined the effects of histamine on expression of glutamate transporter-1 (GLT-1) in an adult rat model of MCAO and found that inhibition of GLT-1 expression reduces excitatory toxicity (42). Notably, the role of excitotoxicity in CVT has not been proven and needs to be further investigated.

#### Metabolic abnormalities

Mitochondria are important organelles involved in energy metabolism (43). ATP produced by mitochondria cannot maintain the energy balance of neurocytes during ischemia and hypoxia after stroke, resulting in cell death (44). Moreover, mitochondrial homeostasis depends on mitophagy and the balance between mitochondrial fission and fusion (45). Grohm et al., reported that mitochondrial fission cause neuronal death after ischemic stroke and that inhibition of Drp1, a regulator of mitochondrial fission, protects neurons from glutamate excitotoxicity and reduces the infarct volume in a mouse model of transient focal ischemia (46). Importantly, on the basis of an MCAO rat model, peroxynitrite aggravates cerebral injury by recruiting Drp1 to damaged mitochondria to activate mitophagy (47). Therefore, mitochondria are potential therapeutic targets for treatment of ischemic stroke. For example, pramipexole restores neurological function though mitochondrial pathways in ischemia/reperfusion injury, such as reducing mitochondrial ROS and Ca^2+^ levels and improving mitochondrial oxidative phosphorylation (48). However, the tentative nature of impaired mitochondrial metabolism in CVT remains unknown and requires experimental confirmation. In general, there is still a lack of research on metabolic abnormalities in CVT.

#### Inflammation

Inflammation may be triggered by various factors after cerebral ischemia, including vascular obstruction, necrotic cells, and tissue injury (49–51). Different types of cells, cytokines and receptors are all involved in inflammatory processes (27). Neutrophils are among early infiltrators into the ischemic stroke brain, increasing within hours of onset and peaking after 1–3 days (52). Activated microglia can induce an inflammatory response. Hyperactivated microglia produce many toxic substances, such as tumor necrosis factor α (TNF-α), interleukin-1β (IL-1β), interferon-γ, IL-6 and ROS, promoting neuronal death (53). Microglia are targets for IGF-1, and the neuroprotective effects of IGF-1 may be mediated by the down-regulation of inflammatory mediators (54). In addition, studies have shown that expression of proinflammatory cytokines and chemokines is increased after stroke. IL-1 (15), TNF-α (55) and IL-6 (56) play important roles in stroke. It has been shown that Toll-like receptors (TLRs) play a role in the inflammatory response by initiating different downstream inflammatory cascades that cause tissue damage. Therefore, these receptors might mediate brain damage following ischemia (57, 58).

TLR2 and TLR4 overexpression is associated with poor outcome and inflammatory response in acute ischemic stroke, and TLR4 is also associated with infarct volume. Importantly, as TLR2- and/or TLR4-neutralizing antibodies impair the induced increase in expression of inflammatory markers in cultured serum cells, TLRs can be regarded as therapeutic targets for ischemic stroke (59). Similar results have been obtained in animal experiments (60).

Additionally, several inflammatory factors play an important role in the development of CVT. As demonstrated by van Aken et al., IL-8 concentrations in plasma above the 90th percentile lead to a 1.9-fold increased risk of venous thrombosis (61). Akbari et al. found that the plasma level of IL-6 in patients with cerebral venous sinus thrombosis was significantly higher than that in patients without thrombosis (62). In addition, proinflammatory cytokines such as IL-6 and IL-8 were found to be elevated in patients with idiopathic venous thrombosis (63). However, the role of inflammation in brain injury after CVT requires further research.

#### Ca^2+^ overload

Ca^2+^ overload has been proven to be involved in the neurotoxic effects of excitotoxicity (64) and oxidative stress (65). Zheng et al. have demonstrated that platelet-derived growth factors counteract the neuroprotective effects of oxidative stress by inhibiting Ca^2+^ overload (66). Moreover, hypoxia can alter intracellular Ca^2+^ channels, such as the Na^+^/Ca^2+^ exchanger, L-type voltage-dependent Ca^2+^ channel, and inositol triphosphate receptor (IP3R) (16). Compensation with miR-132 attenuates the hypoxia-induced increase in Na^+^-Ca^2+^ exchanger 1 (NCX1) expression and decreases apoptosis in cardiomyocytes by preventing Ca^2+^ overload (67). Li et al. reported the beneficial effects of IP_3_R deletion in neuronal protection and reduction of cerebral dysfunction after stroke through disruption of Ca^2+^ signaling in astrocytes (68). The mechanism of Ca^2+^ overload in CVT remains elusive.

#### Cerebral edema and BBB disruption

Both arterial stroke and CVT are associated with disruption of the BBB and edema of the brain (69–71). Brain edema can be divided into three types based on severity: cytotoxic edema, ionic edema, and vasogenic edema (72, 73). Following ischemic injury, cytotoxic edema caused by adenosine triphosphate depletion disrupts Na^+^/Ca^+^ and/or Na^+^/K^+^ channels, resulting in intracellular cation accumulation to equalize ion concentrations without disrupting the BBB (72, 74, 75). Ionic edema, also known as iatrogenic cerebral edema, occurs before the BBB is damaged, and the main sources of edema in the peri-infarct region are the blood and cerebrospinal fluid (72, 76, 77). Furthermore, vascular-derived brain edema occurs at the end of the ischemic cascade. Neuronal death and/or damage caused by cerebral ischemia results in the production of reactive oxygen species, activation of immune cells and release of inflammatory factors, thereby breaking the BBB. After peripheral immune cells invade the brain parenchyma through the BBB, secretion of proinflammatory factors and permeability of the BBB increase, resulting in vasogenic edema (78, 79).

## Molecular pathological hallmarks of hemorrhagic stroke

Hemorrhagic stroke (HS) presents more abruptly and induces more severe complications than ischemic stroke (1). The following discusses critical pathophysiology mechanisms in intracerebral hemorrhage after an arterial hemorrhagic stroke, such as oxidative stress (OS), inflammation, iron toxicity, and thrombin formation (80). Based on this, we speculate on the pathophysiology of venous hemorrhagic stroke.

### Oxidative stress

Oxidative stress has been increasingly acknowledged as having an essential role in secondary brain injury following hemorrhagic strokes (80). Blood cell decomposition products, for instance, iron ions, heme, and thrombin can cause brain damage by producing free radicals (34, 81). Secondly, inflammatory cells, such as neutrophils and microglia, can produce free radicals after hemorrhagic strokes (82). During the inflammatory response triggered by hemorrhagic strokes, neutrophils become stimulated and activated, activating the respiratory chain, and releasing profound ROS, nitric oxide, and so on (83).

### Inflammation

Physiologically, microglia and macrophages regulate the surrounding microenvironment and promote the stability of BBB, neurons, and matrix. When cerebral hemorrhage strokes, excessive microglia and macrophages release numerous inflammatory factors and trigger inflammatory cascades, resulting in pathological changes like BBB injury, edema, and cell death (80). Venous hemorrhagic stroke is not excluded. Following CVT, activated microglia release cytokines, resulting in brain injury, including disruption of the BBB, cerebral venous infarction, and brain edema (84). Immune cells are intensely activated, particularly microglia; macrophage activity increases are proven by Rashad et al. (71). Inflammation plays an essential role in venous hemorrhagic stroke injury, but further research is required.

### Cytotoxicity of erythrocyte lysates

Within 24 hours of a cerebral hemorrhage, large amounts of hemoglobin-containing red blood cells leak into the brain's parenchyma, where they are broken down, which causes hemoglobin to disintegrate into heme and iron is a significant contributor to brain injury affected by hemorrhagic stroke (81, 85). Inflammation, oxidation, nitric oxide scavenging, and edema are the primary mechanisms for brain injury caused by erythrocyte lysates (80). Firstly, HO-1, the critical enzyme for heme degradation, is expressed primarily in microglia after intracerebral hemorrhage and may further exacerbate brain damage by activating microglia and accumulating iron (85). Secondly, free radicals generated by iron may also cause tissue damage. Yeatts et al. confirmed that the iron chelator deferoxamine mesylate has multiple neuroprotective effects, including the reduction of perihematomal edema and neuronal damage, and enhances functional recovery after experimental intracerebral hemorrhage (86). Thirdly, hemoglobin depletes nitric oxide rapidly, triggering microthrombosis in subarachnoid hemorrhage and leading to brain damage (87). Finally, Wang et al. used the intracranial hemorrhage rat model to evidence that hemoglobin and its decomposition products are leading causes of edema (88). All in all, reducing iron accumulation and erythrocyte lysate toxicity is valuable in treating arterial hemorrhagic stroke; however, the same mechanism should be applicable for venous hemorrhagic stroke, but more research is needed to confirm it.

### Thrombin formation

Earlier animal studies demonstrated that intracerebral injection of whole blood rendered brain damage, whereas injection of an inert substance did not produce this effect (89). Furthermore, whole blood injections induce brain injury within 24 hours, as opposed to concentrated blood cells, serum, or unclotted blood plasma (90). Similarly, intracerebral infusions of unheparinized blood results in perihematomal edema formation, while heparinized blood injections do not (91). These findings support the hypothesis that coagulation cascade and clotting may induce brain injury following HS. Thrombin, a prominent part of the coagulation cascade, produces immediately after ICH induction in the brain (92). Thrombin's poisonous or protective effects differ depending on its concentration; infusion of large amounts of thrombin directly into the brain produces inflammation, increased mesenchymal cells, brain edema, scar tissue, and seizures (93, 94). Brain impairments such as cerebral edema and BBB destruction may also occur in venous hemorrhagic strokes. We speculate that thrombin formation may also participate in CVT.

## The clinical heterogeneity of cerebral venous thrombosis

CVT is a specific subtype of stroke with heterogeneous clinical manifestations. In the following sections, we describe the epidemiology, etiology, risk factors, pathological damage, clinical manifestations, diagnosis, treatment, and prognosis of CVT.

### Epidemiological characteristics of CVT

Stroke is a significant cause of disability and vascular death worldwide (95), and ~85% of strokes in adults are ischemic (96). According to a report from the American Heart Association published in 2021, the prevalence of stroke in adults in the United States is 3.4%; the global average lifetime stroke risk rose to 24.9% in 2016 and continues to rise (97). The incidences of CVT and ischemic stroke reported in several studies vary. CVT is an uncommon cerebrovascular event that accounts for 0.5–1% of all strokes in adults (98). At present, there are few epidemiological studies on CVT worldwide, and its true incidence is unknown. According to recent studies in the Netherlands and Australia, the incidence ranges from 13.2 to 15.7/1,000,000 annually (4, 99, 100).

### Special etiology and risk factors for CVT

Risk factors for stroke can be classified as modifiable or nonmodifiable. In general, risk factors for CVT and ischemic stroke have different characteristics (101, 102). Numerous case–control and cohort studies have shown that age, sex, race/ethnicity, and genetics are unmodifiable risk factors for stroke. Thus, there are significant differences in the distribution of the affected population (103–105). Based on a retrospective cohort study of 162 patients, we conclude that CVT primarily affects young adults and children, with a mean age of onset of 42 (±17) years; 70% of patients were younger than 50 years, and 72% were female (106). American Heart Association data indicate that the incidence of ischemic stroke increases with age, and women have a greater lifetime stroke risk than men (107). Sex differences exist because women have specific risk factors, such as oral contraceptive use, pregnancy or puerperium, and hormone replacement therapy (108). A retrospective cohort study by Otite et al. indicated that the incidence of CVT differs by race (Blacks: 18.6–27.2; Whites: 14.3–18.5; Asians: 5.1–13.8) (105). In addition, among 3,298 Northern Manhattan Study participants, Blacks had the highest incidence of stroke, followed by Hispanics and Whites. Thus, stroke is more common in Blacks (hazard ratio (HR) = 1.51, 95% confidence interval (CI), 1.13–2.02) (109).Studies of genetic etiology provides important new insights into the pathophysiology of CVT (110). A genome-wide association study based on 882 patients with CVT, and 1,205 ethnicity-matched controls identified an association with 37 single nucleotide polymorphisms within the 9q34.2 region, this region more than doubled the likelihood of CVT, a greater risk than any previously identified genetic risk marker for thrombosis (111).

**Figure 2:**
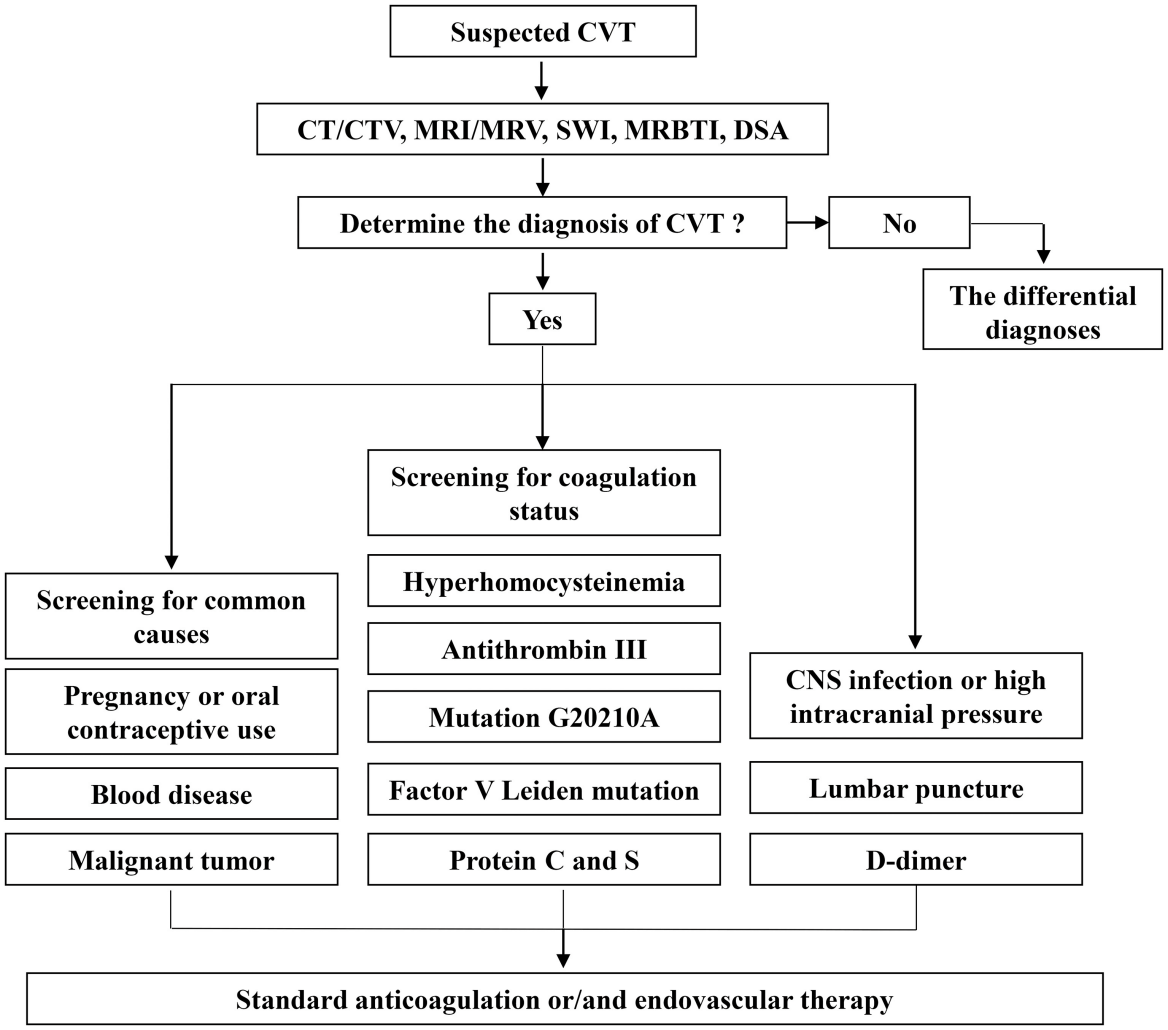
Clinical diagnosis and treatment of CVT.

Different subtypes of stroke are influenced by different risk factors. Regarding risk factors for CVT, in a 12-year retrospective analysis of 83 patients with CVT, 24.1% had infection-associated CVT, with cavernous sinus thrombosis being the most common cause (112). As demonstrated in a case–control study involving 6278 controls and 594 patients with CVT, the risk of CVT in cancer patients was higher than that in those without cancer (odds ratio (OR) = 4.86; 95% CI = 3.46-6.81) (113). Additionally, patients with hematologic cancer have a significantly higher risk (114). The International Study of CVT (ISCVT) also identified lumbar puncture as a risk factor (115); head trauma and surgery are significant factors that should not be ignored (116). Bechchet's disease (9.4%), systemic lupus erythematosus (1.4%), antiphospholipid syndrome (0.6%), iron deficiency anemia (3.2%), ulcerative colitis (*n* = 2) and dehydration (*n* = 3) were risk factors for CVT in a multicenter study of 1,144 patients with cerebral venous thrombosis (117). A meta-analysis conducted by Dentali et al., reported odds ratios for Factor V Leiden mutation of 3.38 (95% CI, 2.27 to 5.05), mutation G20210A of 9.27 (95% CI, 5.85 to 14.67) and hyperhomocysteinemia of 4.07 (95% CI, 2.54 to 6.52) (118). Protein C deficiency increases the risk of CVT by 10.7-fold (3.1–37.7), protein S deficiency by 5.7-fold (1.4–22.4) and antithrombin deficiency by 3.8-fold (1.0–13.8) (119) (Figure 2).

Studies of risk factors associated with ischemic stroke are shown below. According to a worldwide meta-analysis that included 17,663 patients from 32 cohorts in 29 countries, the most important risk factor for stroke is hypertension (Blacks is 52.1%, Asian is 46.1%) (120). In the Northern Manhattan Study, the risk of ischemic stroke was associated with the duration of diabetes (adjusted HR = 1.03 per year with diabetes; 95% CI, 1.02–1.04), and patients with diabetes for more than 10 years had three times the risk compared with those without diabetes (121). The Oxford Vascular Study showed that the incidence of atrial fibrillation associated with ischemic stroke increased with age (122). In a prospective study conducted on individuals without a history of stroke, transient ischemic attack, or coronary heart disease, the researchers found that low-density lipoprotein cholesterol (LDL-C) was positively associated with ischemic stroke. Furthermore, lowering LDL-C to 1 mmol/L with statins may reduce the risk of ischemic stroke (123). The relative stroke risk for one cigarette a day is 1.25 (1.13–1.38) for men and 1.31 (1.13–1.52) for women (124).

### Intracranial hypertension caused by venous thrombotic obstruction is characteristic pathological damage in CVT

Venous return obstruction may result from thrombosis of the cortical cerebral veins, deep cerebral veins, or dural venous sinuses. In contrast to isolated cerebral venous cortical thrombosis, most cortical venous thrombosis occurs in combination with dural venous sinus thrombosis (125). Deep cerebral vein thrombosis generally involves the intracerebral veins and the Galen veins. Approximately 60% of cerebral venous sinus thrombosis (CVST) patients have multiple dural venous sinuses (4). A multicenter clinical study of CVST in 624 patients found the superior sagittal sinus (62%), transverse sinus (41.2–44.7%), straight sinus (18%), and cavernous sinus (1.3%) to be the most commonly affected sites (126). The consequence of venous cerebral infarction is that venous pressure increases, capillary perfusion pressure decreases, cerebral blood volume increases, and intracranial pressure increases (127). Headache is a common symptom in the acute stage of cranial hypertension after cerebral vein occlusion. This is typically a sharp or pulsing pain through the head, both the forehead and top of the head. The headache can be aggravated by coughing, bending, the Valsalva maneuver and elevated cranial hypertension after exertion or even lying down (70). In addition to headaches, visual impairment can manifest, including visual field defects and optic papilledema (128). Symptoms of visual impairment may include swelling, elevation, and blurring of the optic disk, followed by bruising, hemorrhage and even retina infarction, which are due to increased pressure of cerebrospinal fluid in the optic nerve sheath and stagnation of the axoplasmic flow of nerve fibers (129).

It is also worth noting that infarction and hemorrhage are the most significant determinants of neuronal damage and patient prognosis (130). Compared with arterial thrombosis, venous thrombosis is associated with a tendency toward more frequent bleeding due to increased venous and capillary pressure after venous obstruction. Approximately 10–50% of patients with venous occlusion have combined infarction and hemorrhage, mostly at the gray–white matter-cortical junction (131). There is a direct relationship between arterial cerebral occlusion and thrombosis. Arterial cerebral occlusion causes irreversible damage, and imaging typically reveals a small penumbra, whereas venous cerebral occlusion involves unbalanced thrombosis and thrombolysis, yet most regions of the brain are only functionally or metabolically affected and not permanently damaged (132).

Vascular malformation as cause of venous hypertension (133). Dural arteriovenous fistula (DAVF) is a kind of vascular malformation characterized by an abnormal connection between an artery and vein within the dura (134). A reopening of preexisting physiological arteriovenous channels or hypoxia-induced stimulation of neoangiogenesis by venous hypertension has been proposed as pathogeneses of DAVF resulting from CVT (133, 135, 136). Lindgren et al. demonstrated that DAVF occurred in ~2% of CVT patients and was correlated with chronic CVT onset, aging, and male gender according to the data from the international cerebral venous thrombosis consortium (137). Arteriovenous malformations (AVM)' pathogenesis resembles that of DAVF, the relationship of CVT with AVM scarcely has been reported (138).

### Intracerebral hemorrhage and infarction of CVT

Cerebral edema and increased intracranial pressure may develop; thus, hemorrhagic, and ischemic lesions cannot be avoided (139). Venous hemorrhagic stroke-related intracranial hemorrhage appears inhomogeneous surrounded by irregular margins and occurs frequently in the parietal and parietooccipital brain regions adjacent to the cortical and subcortical layers (140). In the VENOST study, of 1,193 patients with CVT, 198 patients had hemorrhagic infarction, 43 patients had intracerebral hemorrhages, acute mode of onset was prominent, neurological symptoms included epileptic seizures (46.9%), altered consciousness (36.5%), nausea and vomiting (36.5%), and focal neurological deficits (33.6%) (*p* ≤ 0.001) (141). In approximately one-third of CVT patients, intracerebral hemorrhages are associated with poor prognoses and severe presentation (141, 142).

### Clinical manifestations of CVT

There are also differences in the clinical manifestations of arterial stroke and CVT (143, 144), with the clinical manifestation of ischemic stroke depending on the site of thrombosis. For example, lesions in the anterior cerebral artery involve symptoms of urinary incontinence, apraxia of gait and motor mutism; lesions in the middle cerebral artery may include hemianopia, impaired movement of arms and legs, aphasia and inattention; lesions in the vertebrobasilar artery are associated with hemianopia, brainstem cranial nerve palsy, ataxia, nystagmus and hemiplegia; and lesions in the small blood vessels are related to lacunar stroke syndrome (145).

In addition to the clinical manifestations of stroke similar to those of arterial stroke, CVT involves high cranial pressure and specific clinical manifestations (117). The most common clinical manifestation of CVT is cranial hypertension, as represented by headache and visual impairment (146). Headache is associated with CVT in at least 85% of patients (147). It usually presents as acute or pulsating pain in the holocranial, forehead, or vertex, which may be isolated or accompanied by other signs or symptoms (148). Visual impairment, including visual acuity impairment, visual field defects and optic papillary edema (129). Acute optic papillary edema was present in 28% of patients during the ISCVST study (126). In the VENOPORT study, 13% of patients had visual impairment, and 2% had significant vision loss (149). Epilepsy (150), psychological and cognitive impairment (151), and dural arteriovenous fistula (152) are also specific clinical manifestations of CVT.

### Challenges in the diagnosis of CVT

The clinical and radiological characteristics of CVT are nonspecific, which delays diagnosis and subsequent treatment (153). Therefore, cases of CVT have a high rate of under- and misdiagnosis, and the median time from onset to diagnosis is ~7 days (144, 154). On the contrary, as ischemic stroke is a condition with a narrow treatment window, rapid diagnosis and prioritization are necessary. Computerized tomography (CT)/computerized tomography venography (CTV) and magnetic resonance imaging (MRI)/magnetic resonance venous imaging (MRV) can be used as the preferred examination methods for arterial and CVT. Digital subtraction angiography (DSA) is the gold standard for both diagnosis (4, 155) (Figure 2). Aside from imaging studies, the necessary hematology, coagulation, and biochemical tests should be performed (4) (Figure 2). In a prospective study of 34 patients with acute CVT, other auxiliary tests, such as D-dimer levels, had a sensitivity of 94.1% and specificity of 97.5% for the diagnosis of stroke (156).

#### Cranial computed tomography/computed tomography venography

The direct signs of CVT on noncontrast CT are often referred to as the “dense clot sign” or “cord or string sign,” that is, the high-density shadow of thrombi in the cerebral sinus and veins (157). Within two weeks, the density of the thrombus gradually declines to the average level (158). Contrast-enhanced CT help assess the venous sinuses and cortical veins filling defects, changes in collateral venous drainage, and the vein (sinus) walls (159, 160). The specific sign on contrast-enhanced CT is called the “empty delta sign,” indicating superior sagittal sinus thrombosis (161). Hemorrhagic infarction, brain edema and mass effect are common indirect CT signs, which are more common than direct signs (162). A meta-analysis indicated a sensitivity of 0.79 and specificity of 0.90 for CT (163). CTV can diagnose cerebral sinus thrombosis accurately, but its use in the diagnosis of cortical vein thrombosis is limited (164). The preferred diagnostic modalities for ischemia are CT and MRI, CT with sensitivities of 57–71% in the first 24 h compare to MRI (165). In addition to assessing acute ischemic stroke, noncontrast CT can be used to evaluate acute infarct size. The method for quantifying the size of the infarct is the Alberta Stroke Programme Early CT Score (166, 167). CT angiography is the first choice to detect intracranial large vessel occlusion, with a sensitivity approaching 100% (168).

#### Cranial magnetic resonance imaging /magnetic resonance venous imaging

MRI and CT can show the same direct and indirect signs, but MRI has advantages in comparison to CT in detecting parenchymal lesions and cerebral edema (169). Thrombus appearances on different MRI sequences are dependent on the time of evolution (170). On T1-weighted images, it appears isointense within 5 days; on T2-weighted images, it seems hypointense. The thrombus becomes hyperintense on both T1 and T2 sequences within 6–15 days. After 15 days, they turn isointense on T1 and iso- or hyperintense on T2 sequences. Upon examination of T1 and T2 sequences four months later, no abnormalities were detected (170). Diffusion-weighted imaging can distinguish between vasogenic and cytotoxic oedemas (152). A study of 23 patients with cerebral venous thrombosis confirmed by the novel magnetic resonance black-blood thrombus imaging (MRBTI) method showed that MRBTI can be successfully used as first-line diagnostic imaging (171, 172). Time-of-flight MRV (TOF-MRV) and contrast-enhanced MRV(CE-MRV) are two of the most frequently used MRV techniques. CE-MRV provides better visualization of cerebral venous anatomy without being dependent on blood flow signals, therefore, more sensitive than TOF-MRV, but less sensitive to isolated cortical venous thrombosis (161, 173). Compared with CT, MRI has a sensitivity of 73–92% within 3 h and close to 100% within 6 h (168). Based on a prospective study of 267 patients, MRI was more sensitive than CT in diagnosing acute ischemic stroke with large vessel occlusion (174).

#### Digital subtraction angiography

Ultimately, DSA is the gold standard for diagnosis (175). Nevertheless, with the development and widespread application of imaging technology, invasive DSA is rarely required to diagnose CVT. DSA is recommended if the non-invasive imaging examination is uncertain, endovascular treatment is considered, or DAVF is suspected (4).

### Treatment of CVT

#### General treatment

The first step in the treatment of CVT is to actively treat the primary disease. If anticoagulation is not contraindicated in patients with CVT, it should be performed as soon as possible, and low molecular weight heparin should be used in the acute phase (4) (Figure 2). This view has been confirmed by a meta-analysis involving 79 patients (176). The oral anticoagulant warfarin should be taken after the acute phase. Direct oral anticoagulants (DOACs) like dabigatran are most likely to provide benefits in treating CVT (177). Among 845 CVT patients in a multicenter international retrospective study, 33.0% received DOACs alone, 51.8% received warfarin alone, and 15.1% received both treatments simultaneously (178). Compared with warfarin treatment, DOACs were linked with an analogous risk of recurrent venous thrombosis (aHR, 0.94; *P* = 0.84) but a lower risk of significant hemorrhage (aHR, 0.35; *P* = 0.02) (178). Another international retrospective cohort study of 766 patients with CVT, showed an overall incidence of 35.1 recurrences per 1,000 patient-years (95% CI, 27.7–40.4) after discontinuation of anticoagulant therapy, indicating that oral anticoagulants are effective at reducing the recurrence and mortality of CVT (179). Nevertheless, the efficacy of new oral anticoagulants remains to be further observed.

#### Endovascular therapy

Endovascular therapy has therapeutic value for both venous and arterial stroke (180, 181). Anticoagulation is not always effective in patients with CVT, so endovascular treatment (EVT) may be beneficial for these patients (182) (Figure 2). In a systematic review of 26 patients, local thrombolysis was found to be beneficial but associated with a certain risk of bleeding (183). Thrombolysis or Anticoagulation for Cerebral Venous Thrombosis (TO-ACT) studied the use of neuro intervention vs. conventional treatment in patients with severe CVT (characterized by deep venous involvement, intracranial hemorrhage, Glasgow coma score (GCS) < 9), 67 participants were randomized, the trial was terminated early for futility due to no difference in the modified Rankin Score(mRS) at 12 months (67 vs. 68%; RR 0.99, 95% CI 0.71–1.38) (184). EVT has not yet been proven effective in patients with CVT based on the available evidence, the data of EVT in CVT are derived from some small retrospective studies, patients weren't assigned randomly, and the study was probably influenced by disease severity, thus prone to bias (184–186).

### Prognosis of CVT

The overall prognosis of CVT is favorable. The VENOST study of 1,144 patients with CVST showed that 78.4% had a modified ranking scale (mRS) of 0–1, 11.7% had an mRS of 2, and 10.0% had an mRS of 3–5 (117). The ISCVST study followed CVST patients for 6 months after discharge and found that 78.1% recovered completely, with an mRS of 0–1, 8.0% had a partial recovery, with an mRS of 2, and 14.0% had functional disability or died, with an mRS of 3–6 (187). Ischemic stroke is incurable, has a poor prognosis and is often accompanied by complications. A total of 76.9% of patients had at least one complication, and 20% experienced three or more (188). A cohort study of 1,075 patients who underwent rehabilitation after stroke in Poland confirmed that the most common complication of ischemic stroke is urinary tract infection (23.2%), followed by depression (18.9%), falls (17.9%), unstable hypertension (17.6%) and shoulder pain (14.9%) (188).

## Conclusion

As a particular type of stroke, CVT is usually considered a disease with favorable outcomes, mostly occurring in young and middle-aged patients; however, at least 13% of all patients die or are severely handicapped. The traditional pathophysiological mechanisms of strokes have focused on the results due to artery thrombosis and fail to deeply explore the process and results of cerebral venous thrombosis. Compared with arterial stroke, cerebral venous thrombosis characterized by intracerebral hemorrhage with infarction, which might be termed as venous stroke needs to be further studied.

## Author contributions

YZ and HJ: design the article structure and write the paper. XJ and CZ: propose research ideas, design the article structure, check the overall situation, and approve the paper. HW and LL: research and organize the literature and verify the paper. All authors contributed to the article and approved the submitted version.

## Funding

This study was supported by Pharmaceutical Collaboration Project of Beijing Science and Technology Commission (Z181100001918026), and National Natural Science Foundation of China (82271311).

## Conflict of interest

The authors declare that the research was conducted in the absence of any commercial or financial relationships that could be construed as a potential conflict of interest.

## Publisher's note

All claims expressed in this article are solely those of the authors and do not necessarily represent those of their affiliated organizations, or those of the publisher, the editors and the reviewers. Any product that may be evaluated in this article, or claim that may be made by its manufacturer, is not guaranteed or endorsed by the publisher.
